# Record-Breaking Pain: The Largest Number and Variety of Forelimb Bone Maladies in a Theropod Dinosaur

**DOI:** 10.1371/journal.pone.0149140

**Published:** 2016-02-24

**Authors:** Phil Senter, Sara L. Juengst

**Affiliations:** 1 Department of Biological Sciences, Fayetteville State University, Fayetteville, North Carolina, United States of America; 2 Department of Anthropology, Appalachian State University, Boone, North Carolina, United States of America; Faculté de médecine de Nantes, FRANCE

## Abstract

Bone abnormalities are common in theropod dinosaur skeletons, but before now no specimen was known with more than four afflicted bones of the pectoral girdle and/or forelimb. Here we describe the pathology of a specimen of the theropod dinosaur *Dilophosaurus wetherilli* with eight afflicted bones of the pectoral girdle and forelimb. On its left side the animal has a fractured scapula and radius and large fibriscesses in the ulna and the proximal thumb phalanx. On its right side the animal has abnormal torsion of the humeral shaft, bony tumors on the radius, a truncated distal articular surface of metacarpal III, and angular deformities of the first phalanx of the third finger. Healing and remodeling indicates that the animal survived for months and possibly years after its ailments began, but its right third finger was permanently deformed and lacked the capability of flexion. The deformities of the humerus and the right third finger may be due to developmental osteodysplasia, a condition known in extant birds but unreported in non-avian dinosaurs before now.

## Introduction

Fractures, punctures, and other bone maladies are common in the skeletons of non-avian theropod dinosaurs [1–5]. The pectoral girdle and forelimb are frequently afflicted, which suggests vigorous use of the forelimbs [3,4]. Only six non-avian theropod skeletons are known to have pathological features on more than one bone of the pectoral girdle and/or forelimb. In four of the six specimens, only two pectoral girdle and/or forelimb bones are known to be afflicted. A specimen of *Allosaurus fragilis* bears an idiopathic lesion on the right scapula and a fractured and infected proximal phalanx of the right second finger [6], a specimen of *Deinocheirus mirificus* bears evidence of injury on the proximal two phalanges of the left third finger [7], a specimen of *Tyrannosaurus rex* exhibits a collapsed glenoid with deformation of parts of the left scapula and coracoid [8], and another specimen of *T*. *rex* exhibits a furcula with a stress fracture and a left humerus with extensive periostitis apparently resulting from a tendon avulsion [8]. A third specimen of *Tyrannosaurus rex* bears pathological features on four pectoral girdle and forelimb bones. It exhibits a fractured furcula, an exostosis on the right coracoid, a possible tendon avulsion on the right humerus, and a deep pit on the right first metacarpal that may be due to gout [4,8–10]. Before now, this was the highest number of pectoral girdle and/or forelimb bones reported to bear pathological features in a non-avian theropod dinosaur. Here, we report the presence of twice this number of afflicted pectoral girdle and forelimb bones in a non-avian theropod dinosaur, *Dilophosaurus wetherilli*.

**Fig 1:**
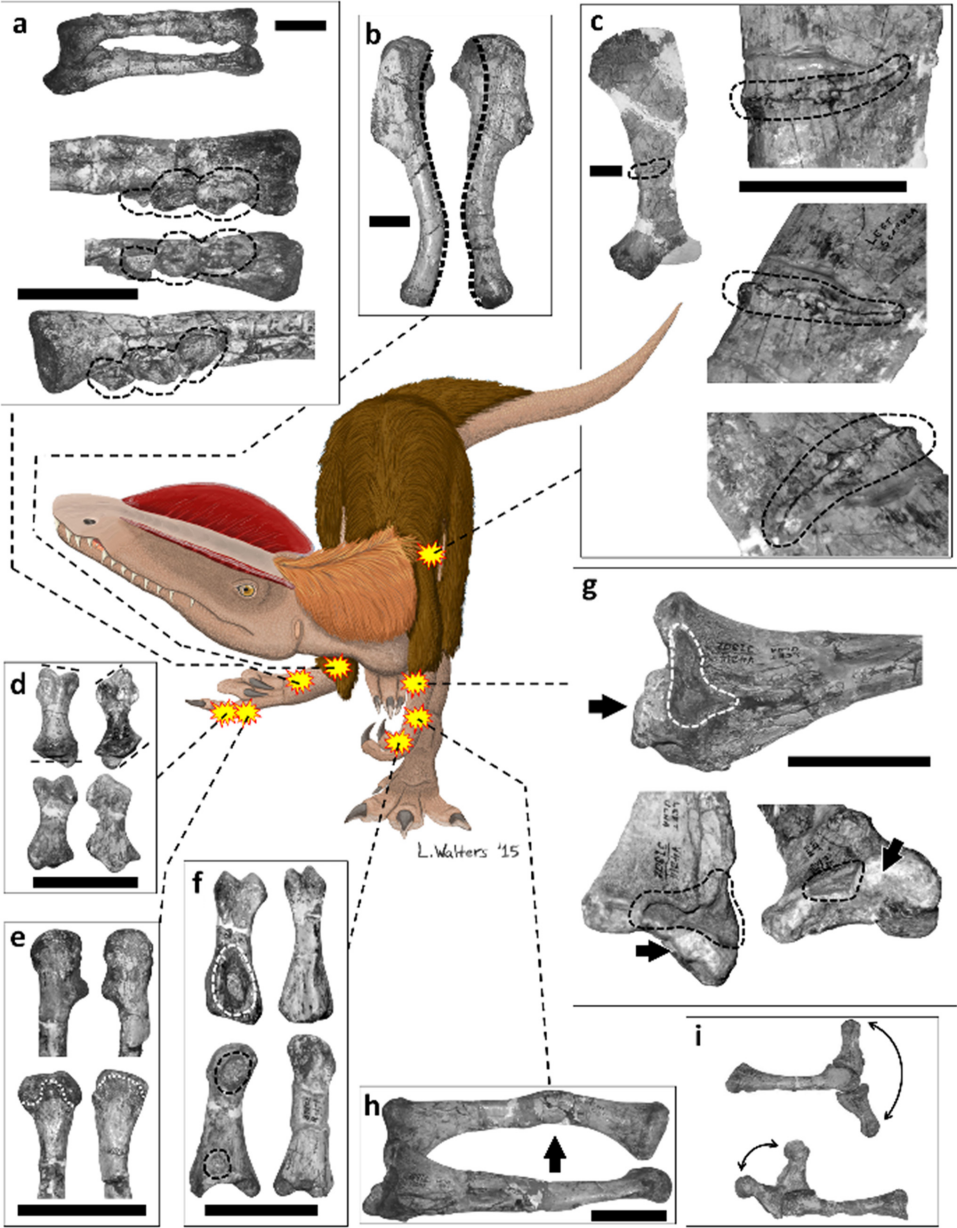
Pathological features in the forelimbs and left scapula of UCMP 37302 (*Dilophosaurus wetherilli*). (a) Right radius and ulna (above) and enlargements of distal end of radius (below) in (from top to bottom) lateral, abductor, and medial views; broken outline indicates three bony tumors. (b) Left and right humerus (left humerus on left, right humerus on right) in lateral view, each photographed with lateral epicondyle directly facing the viewer, with heavy broken line indicating the midline of the posterior (retractor) surface of each to show the abnormal degree of torsion in the right humerus. (c) Medial surface of left scapula, with broken outline indicating fracture. (d) Left (on left) and right (on right) manual phalanx III-1 in dorsal (top) and palmar (bottom) views, with broken lines indicating plane of articulation with adjacent bones, to show the alteration of this plane in the right-hand phalanx. (e) Distal ends of left (on left) and right (on right) metacarpal III in lateral/abductor view (top) and palmar view (bottom), with broken outline indicating edge of articular surface, to show abnormal truncation of articular surface in right metacarpal III. (f) Left manual phalanx I-1 (on left), with its right-hand counterpart for comparison (on right), in palmar (top) and lateral/abductor (bottom) views, with broken outlines indicating healed fibriscesses. (g) Medial surface of left ulna, with broken outline indicating healed fibriscess and arrow indicating abnormal bony growth. (h) Left radius and ulna in medial view, with arrow indicating healed fracture. (i) Left (top) and right (bottom) metacarpal III and phalanx III-1, with phalanx III-1 in full extension and full flexion, to show the reduced range of motion of this digit in the right hand. Scale bars = 50 mm.

*Dilophosaurus wetherilli* is a basal neotheropod dinosaur [11] from the Kayenta Formation of Arizona [12]. The holotype specimen, UCMP 37302, is publicly-deposited and accessible to researchers as part of the collection of the University of California Museum of Paleontology (UCMP) in Berkeley, California. This study involved surface examination of the specimen at the museum. Eight pectoral girdle and forelimb bones bear pathological features in the specimen (Fig 1A–1H).

Table 1 lists symptoms that we used for diagnosis. We used extant reptiles and birds as model organisms when possible, because non-avian dinosaurs are phylogenetically bracketed by extant reptiles and birds and because symptoms of pathological conditions of reptile and bird bones often differ from the symptoms of the corresponding ailments in mammals (Table 1). The use of mammals such as humans as model organisms in diagnoses of pathological conditions in dinosaur fossils is therefore potentially misleading, although it is the only recourse in cases for which corresponding conditions in reptiles and birds have not been sufficiently described.

**Table 1:** Macroscopic, externally-visible features of bones with pathological conditions in extant amniotes.

Condition	Reptilia (non-avian)	Aves	Mammalia
Arthritis			
Gout	lytic lesions in, around, or near joints [16]; spheroidal lytic lesions reported only in dinosaurs [9]	para-articular, spheroidal lytic lesions with a scooped-out appearance [17]	para-articular, spheroidal lytic lesions with a scooped-out appearance on both sides of a joint (especially in the autopodium) [18]
Immunoarthritis	rheumatoid arthritis: R [19]		bilateral osteophytes (bone spurs) adjacent to articular margin; focal areas of bone loss; dislocation in some cases [20].
Osteoarthritis	lysis of articular surfaces, and bony proliferation around margins of articular surfaces [20]	eburnation of joint surface, with osteophytes on rim of articular surface [21]	triangular osteophytes on articular margin [20]; erosion of articular surfaces, with or without lysis and marginal lipping [18].
Osteochondritis (= osteochondrosis)	lysis of subchondral articular surfaces [20]	small, focal lesions; articular surface with irregular margins [17].	opposing joint surfaces dissimilar; bilateral osteophytes adjacent to articular margin [20].
Septic arthritis	lysis at the joint surface, eroding subchondral bone [20,22]	lysis at the joint surface [23], with disorganized texture [17]	osteomyelitis of joint surfaces, *often with ankyloses* [18]
Infection			
Osteomyelitis	General: lysis *without deformation of overall shapes of bones*. [24]. Non-mycobacterial: lysis with *minimal or no periosteal proliferation* [22,25]. Mycobacterial (including tuberculosis): lysis with significant periosteal proliferation [22,26], often with vertebral fracturing [26]. General: *lytic defect often persists after infection has healed* [13]; in extreme cases lysis may obliterate an entire bone; periosteal reaction is stronger in vertebrae than in appendicular bones, and may result in a large callus with fusion between vertebrae [24]	General: lysis with *minimal or no periosteal proliferation* [22] *and without deformation of overall shapes of bones*. [27]. Mycobacterial (including tuberculosis): multiple focal lytic lesions [27]	lysis with involucrum; destructive-productive bone reaction that often produces irregular and bizarre surface texture (e.g. coralization) *and often deformation of bone shapes*; *aggressive periosteal proliferation* often hides the lysis [18,20]; may result in fractures [20,28]
Periostitis		well-circumscribed, local thickening of cortex [17]	local thickening of cortex [20], well-circumscribed and with or without porous or sunburst texture [18]
Metabolic bone disease			
Chondrodystrophy		shortening of long bones; enlargement of ankle joint; varus or valgus leg deformities [29]	
Fibrous osteodystrophy	bones with enlarged diameter [30–32] and irregular surface texture/outline [16,30,33]; underbite [33,34], often with lateral bowing of mandible [16]; fractures of vertebrae and weight-bearing long bones are common [30]	lateral twisting and outward bending of tibiotarsus, often unilateral [29]	increase in cortical thickness; may result in fractures [28]
Osteomalacia	kyphoscoliosis of vertebral column; in some cases, teeth bent into horizontal position [35,36]; *bowed long bones*, thin cortices, increased osteoid deposition at stress points (e.g. tendon insertions, curvatures), folding fractures [32]	deformation of skull bones [27]	widespread deformities of vertebral column and pelvis; may result in fractures of long bones; *long bone deformities usually due to fracturing rather than bending* [18]
Osteoporosis	R (but one reference does mention poor ossification and fracturing of long bones [33])	spinal deformation (e.g. kyphosis, rarely scoliosis) [27]; *deformed sternum* [29,37]; fractures, especially in appendicular bones [37]; *sigmoid deformation of ribs* due to fractures at the joint between vertebral rib and sternal rib [29]	spinal fractures and resulting spinal deformities [18]
Rickets	pronounced widening of articular ends of long bones; spontaneous fractures; often, rachitic rosettes (bulbous swellings of sternal tips of ribs) [20,32]	widening of growth plates, with decreased bone apposition; enlargement of joints; bowing of long bones of legs [38,39]; bending deformation of long bone metaphyses, vertebrae (e.g. kyphosis, rarely scoliosis), ribs, pelvis, and sometimes skull bones [27]; enlargement of epiphyseal region at proximal ends of ribs [29]	bending of long bones; widening of growth plates, *often with cup-shaped depression of distal metaphyseal surface* [18,20]
Secondary hyperparathyroidism	development of rickets or osteomalacia [24,32]; fractures of long bones, pelvis, and mandible [40], without healing; angular deviations of long bones [34]; paresis of hindlimbs [34,40]	hindlimb long bones with normal epiphyses but with deformations of and folding fractures in diaphyses [41]; deformation of ribs, sternum, and pelvic girdle due to fibrous dysplasia [42]; development of rickets [39] or osteomalacia [39,43]	stunted growth and symptoms like those of rickets [18]
Neoplasm			
Osteochondroma	“ballooning” expansion from a bony surface; rounded, bulky, with smooth, often billowy or undulatory margin and lack of discrete cortex in primary tumor growth region [44]		“ballooning” expansion from a bony surface; rounded, bulky (or cauliflower-like in the pelvis), with smooth, often billowy or undulatory margin and lack of discrete cortex in primary tumor growth region [18,20]
Osteoma	small, smooth lump [45]	small nodule [46,47]	small, smooth lump, with or without a small cavitation or multiple lobules [18,48,49]
Osteosarcoma		bony outgrowth [47] that may be characterized by extreme periosteal proliferation [50] that may resemble its mammalian counterpart [27]; may result in fractures [27]	poorly-marginated growth consisting of plumes of cortex extending outward, often in a sunburst or coralized pattern; no involucrum; extreme bone destruction that mimics amputation in some cases [18,20]; may result in fractures [20,28]
Other			
Avulsion of tendon or ligament	cavitation into bone surface, surrounded by spalling of bone from tendon or ligament attachment site (described in dinosaurs [4])	R [51]	spalling of bone from tendon or ligament attachment site [20]
Fracture (other than stress fracture)	break, with or without misalignment; *periosteal callus (if present) much less extensive than in mammals* [12,15,52]	break, with or without misalignment; *periosteal callus (if present) much less extensive than in mammals* [22]	break, with or without misalignment; *periosteal callus extensive*; callus dense with smooth surface in old, healed fractures [18,20]
Osteodysplasia		abnormal torsion of the tibiotarsus or tarsometatarsus (or, less commonly, the femur or phalanges), usually of only one hindlimb, producing *angular deformity without dislocation and with remodeling of joints to retain good fit* [36,38,53,54]; or abnormal torsion of the autopodium of one forelimb [55].	misshapen articular surfaces leading to *poor fit of limb bones at joints* (often the hip joint), *often resulting in dislocations* and fractures; deformation of long bone shafts [20]
Osteopetrosis		thickened cortex; *extreme enlargement of diameter of diaphyses of long bones*, *starting at mid-diaphysis and proceeding toward metaphyses*; the tibia and metatarsus are affected first, followed by other long bones and ribs and bones of the pectoral and pelvic girdles, but not digits [56]	thickened cortex; *enlarged ends of long bones*; often, transverse fractures of long bones [18]. The disease known as osteopetrosis in mammals is genetic [18] and should not be confused with the avian disease of the same name, which is caused by a virus [56].
Pseudarthrosis	functional articular surfaces between two parts of one bone (after fracture) (described in dinosaurs [5])		functional articular surfaces between two parts of one bone (after fracture) or misshapen and between two bones at an abnormal location (after luxation or subluxation) [18,20,57]
Stress fracture	lump on diaphysis (described in dinosaurs [4])		local cortical thickening [45]

Italics indicate differences between reptiles/birds and mammals. This table omits conditions reported only in mammals and conditions that affect only the skull or vertebral column. It also omits symptoms described in snakes, because the responses of bone to trauma or disease in snakes are often more extreme than they are in other reptiles [13–15]. R = recorded but insufficiently described for external, macroscopic comparison with fossil bones.

## Descriptions and Diagnoses

### Left scapula

On the internal surface of the left scapula is an incomplete fracture that extends transversely 5–6 cm from the posterior margin of the scapular blade (Fig 1C). The fracture does not entirely transect the bone but stops approximately 1 cm before the anterior margin. A bony callus is present along the fracture on both sides. The callus is less than 1 cm high, consistent with the minor periosteal reaction that follows avian and reptilian bone fractures [13,15,22] and unlike the aggressive periosteal reaction that follows bone fractures in mammals [18,20]. This callus appears smooth and remodeled, and its maturity suggests that the fracture had healed with good alignment, which further suggests that the fracture occurred at least some weeks before death. The healing rate for fractured bones in non-avian dinosaurs is unknown, but bone fractures typically heal in two to six weeks in extant birds [58]. In extant reptiles traumatic fractures take six to thirty months to heal [15], and fractures from metabolic bone disease take six to eight weeks to heal [59]. The lack of bone lysis indicates that the bone did not become infected following fracture (Table 1).

The specimen’s scapular fracture could have resulted from a violent interaction or a fall. In humans, scapular fractures most often occur from a fall from a height or assault while the victim is prone [60,61]. However, in humans the scapula is on the dorsal surface of the ribcage, whereas in non-avian theropods it is on the lateral surface of the ribcage [62,63]. A better analogue than the human scapula in this case is therefore the ratite forelimb. This is because in extant birds the pectoral appendage is reoriented so that the scapula is dorsal to the ribcage and the forelimb is lateral to the ribcage. The location of the ratite forelimb therefore closely matches that of the non-avian theropod scapula. In farmed ratites forelimb fractures are usually caused by collision with a hard, vertical surface such as a tree or barn, or by kicks from conspecifics [64]. The fracture to this specimen’s scapula therefore may have resulted from impact with a hard, vertical surface or conflict with another animal while the animal was upright, although it also could have resulted from a fall onto its side.

### Left radius

The left radius exhibits a fracture on the middle third of the shaft, as a result of which the shaft is bent at an angle of about 20° toward the ulna a little over halfway down its length (Fig 1H). Remodeling and the angle of the radial shaft show that this fracture was healed. Lack of lysis indicates that the bone did not become infected after the fracture (Table 1). Plausibly, the fracture resulted from accidental trauma such as falling; distal radial fractures in humans are most commonly the result of falls [65,66]. The degree of healing and the presence of the radial and scapular fractures on the same side of the body are consistent with an inference that the two fractures occurred at the same time.

### Left ulna

The left ulna exhibits a large lytic depression and an abnormal bony growth medial to the proximal articular surface (Fig 1G). The depression is on the medial side of the ulna, immediately distal to the humeral articular surface, and is 16 mm in length (parallel to the long axis of the ulna) and 35 mm in height (perpendicular to the long axis of the ulna, in the plane of elbow flexion/extension). The smooth walls of the lesions indicate that the infection was healed by the time of death. The ulnar abnormalities do not represent a fracture; there is no indication of a break in the bone. We interpret the abnormalities of this ulna as osteomyelitis following a puncture wound. In extant reptiles osteomyelitis typically follows the penetration of bone by a puncture. It produces lysis with permanent bone loss at the afflicted area, leaving a permanent cavity even after healing. The relatively small size of the bony growth is consistent with the minor periosteal reaction of osteomyelitis in birds and reptiles [15,22,67].

### Left thumb: proximal phalanx

The palmar surface of the proximal phalanx of the left thumb exhibits a large, smooth-walled, abnormal cavity (Fig 1H and 1F). The floor of the cavity is approximately 7 mm wide and 13 mm long, and its rim is approximately 13 mm wide and 23 mm long.

On the same phalanx there is also a small, pathological pit at the proximal end of the lateral (abductor) surface of the phalanx. In addition, the lateral collateral ligament pit at the distal end is abnormally enlarged (Fig 1F). The deep palmar cavity, the small proximal pit, and the enlargement of the collateral ligament pit appear to be fibriscesses. Fibriscesses in dinosaur bones are often called “abscesses” in the literature, but mammalian abscesses are characterized by pus formation, which is absent in the corresponding pathological features of birds and other reptiles. Here, therefore, we use the recently-coined term “fibriscess” for the reptilian equivalent of mammalian abscesses [68]. The two shallower fibriscesses may represent lysis due to the spread of the infection that entered the phalanx at its presumed puncture wound, the deep fibriscess on the palmar surface. The smooth walls of all three abnormalities on this phalanx indicate that eventually the bone was effectively able to contain and heal the infection [69].

In this set of lytic abnormalities there is no bony spalling as would be present following a tendon or ligament avulsion [4]. The lytic areas’ lack of spheroidal shape does not suggest gout [9, 17], and other forms of arthritis are ruled out by the lack of lysis on articular surfaces and the lack of osteophytes (Table 1). As with the ulnar abnormalities, this set of lytic areas is consistent with osteomyelitis following a puncture. The depth of the proximal cavity on the palmar surface suggests that this was the location of the puncture. Such a puncture does not indicate a bite wound, because there are no tooth puncture marks on any of the other forelimb bones. The palmar surface of the hand faces medially in non-avian theropods [70–73], and the possible puncture wound on the ulna is also on the medial surface. If these abnormalities resulted from the kick (or kicks) of a conspecific, the two individuals must therefore have been facing each other at an angle, with the kicking individual at the victim’s front and right in order to have struck the left forelimb’s medial surface without hitting the right forelimb. Alternately, the assailant may have approached from the victim’s left side and hooked the victim’s left forelimb in its manual claws. Another possible scenario is a set of kicks from a clutched prey item.

### Right humerus

The shaft of the right humerus exhibits approximately 35° more torsion about its long axis than in the left humerus (Fig 1B). Theropods usually exhibit torsion in the humeral shaft, such that when the left humerus is seen in proximal view the condyles are offset counterclockwise, and when the right humerus is seen in proximal view the condyles are offset clockwise. In the *D*. *wetherilli* holotype this torsion is present with typical magnitude in the left humerus. The abnormally high degree of torsion in the right humerus caused the right forearm and hand to protrude laterally at an unusual angle. The humerus exhibits no apparent evidence of any injury or other physical insult that could have caused the hypertorsion. The specimen also lacks the typical avian and reptilian signs of metabolic bone disease: enlarged long bone diameter with irregular surface texture; widening of metaphyses; bowing of long bones; and deformities of the skull and vertebral column (Table 1).

The deformity may be due to a type of osteodysplasia similar to one that afflicts juvenile birds with nutritional deficiencies other than those that cause metabolic bone disease. Afflicted birds keep their weight on one hindlimb to avoid pain in the other hindlimb, and the weight-bearing hindlimb develops torsion of the tibia or metatarsus [36,38,53,74]. In this specimen of *D*. *wetherilli* neither the tibiae nor the metatarsals exhibit abnormal torsion, but it is possible that it suffered from a similar condition of the forelimb and that its humeral deformity resulted from preferential use of the right forelimb to avoid pain in the left. A form of osteodysplasia afflicting only the forelimb, in which abnormal torsion is present in the carpometacarpus of one wing but not the other, is known in extant waterfowl [55].

### Right radius

The distal portion of the right radius has three bony tumors, arranged in a proximodistal row, on the surface facing the ulna (Fig 1A). The proximal tumor is 19 mm long, 17 mm high (in the dimension of elbow flexion/extension and wrist abduction/adduction), and 11 mm wide (in the dimension of wrist flexion/extension). The middle tumor is 18 mm long, 10 mm high, and 9 mm wide. The distal tumor is 23 mm long, 9.5 mm high, and 9 mm wide.

It is not possible to determine the etiology of these tumors at present. A similarly blocky growth on a mosasaur vertebra was identified as an osteoma (benign bony tumor) [75], but osteomas are smaller, smoother, and rounder than that growth and the growths on the *D*. *wetherilli* radius [45]. It is possible that they represent a malignancy (osteosarcoma), but this is not certain. Their morphology does not match that of mammalian osteosarcoma. Osteosarcoma is undescribed in reptiles, and in birds it may match its morphology in mammals (Table 1). A full diagnosis of these tumors therefore awaits data from future studies.

### Right metacarpal III and phalanges of digit III

**Fig 2:**
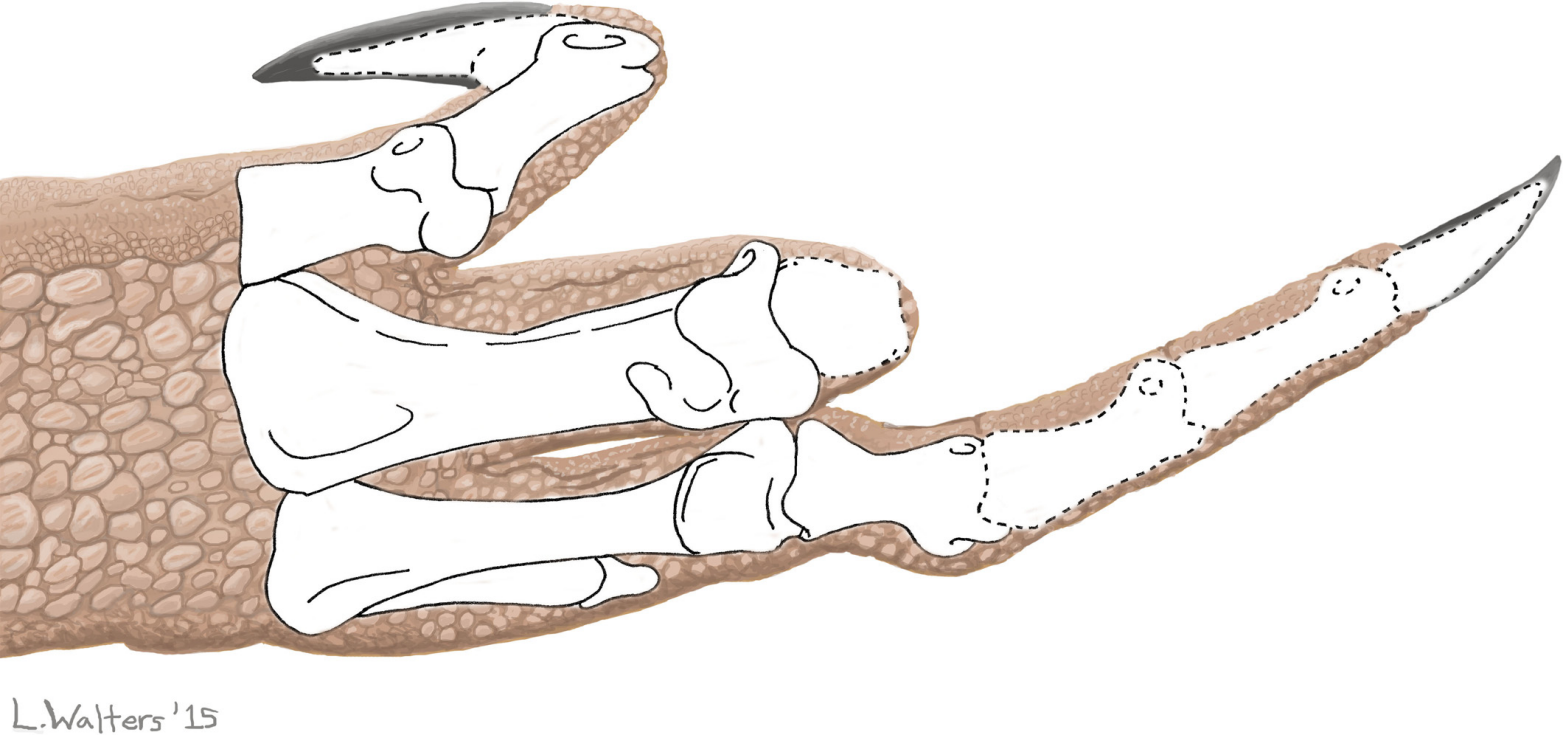
Hand of UCMP 37302 (*Dilophosaurus wetherilli*) in full flexion, showing pathological orientation of the phalanges of finger III. Note that the third finger is abnormally angled in two places: at the metacarpophalangeal joint and at the first interphalangeal joint. Bones with broken outlines are missing from the right hand and are reconstructed according to their shapes in the left hand.

On metacarpal III of the right hand the distal articular surface is truncated at its palmar end (Fig 1E), reducing the range of motion of the proximal phalanx and restricting it to extension and hyperextension, with no capability of flexion (Fig 1I). The proximal phalanx of this digit is misshapen. Its proximal articular surface is slanted such that the lateral (abductor) side is distally displaced, which set the finger at a permanently-abducted angle instead of allowing the finger to project straight out distally from the metacarpal. The distal articular surface is also slanted such that the lateral (abductor-side) condyle is distally displaced, which set the second phalanx at a permanently-adducted angle instead of allowing it to project straight out from the first phalanx (Fig 2). The finger was therefore not only permanently extended/hyperextended but also oriented at an abnormal angle at two joints. At the joint between the metacarpal and the proximal phalanx the misshapen articular surfaces of both bones fit each other perfectly, so that through the arc of the remaining range of motion the two bones are in complete articulation, and the phalanx glides smoothly over the distal articular surface of the metacarpal (Fig 1I). Through most of this arc of motion the palmar surface of the proximal phalanx is displaced toward the other digits rather than being oriented toward the palm.

The cause of the deformity in this finger is difficult to determine, but some potential causes can be ruled out. Arthritis is ruled out by the absence of lysis, osteophytes, and other indicators of arthritis (Table 1). The absence of deformities in bones of the skull, vertebral column and hindlimbs rules out the various forms of metabolic bone disease (Table 1) as the source of the apparent skeletal plasticity in the specimen’s right forelimb.

The abnormal torsion of the right humeral shaft kept the forearm and hand oriented outward so that the hand could not be tucked in toward the trunk. This placed the distal forelimb at greater risk of injury than it normally would have been. It is therefore conceivable that the deformities of the right third metacarpal and finger are the result of metacarpophalangeal and interphalangeal dislocations that occurred during the realization of this risk, with subsequent formations of pseudarthroses that allowed continued articulation of these bones. However, the perfect fit of the bones with each other, such that each glides smoothly over the other during manual manipulation, suggests that the bones were never dislocated but instead experienced angular deformities while remaining in articulation.

If the abnormal torsion of the humerus was a result of osteodysplasia, then it is plausible that the angular deformities in these phalanges were due to the same condition. In extant birds with osteodysplasia, affected limb joints undergo angular deformity but maintain goodness of fit between the bones [36,38,53,54], as in the bones of this dinosaur’s third finger.

It is also possible that the unusual angulation within the right third finger is related to preferential use of the right forelimb to avoid pain in the injured and infected left forelimb. In such a case, without compensatory use of the left forelimb to balance the loading of forces between the two forelimbs, the right forelimb would have experienced loading at unusual angles, which would have been made extraordinarily unusual by the abnormal orientation of the distal forelimb due to abnormal humeral torsion. The resulting metacarpal and phalangeal remodeling would have caused at least some deformation even without osteodysplasia, but the high magnitude of angular deformity in these bones suggests a contribution by osteodysplasia.

## Discussion

This specimen’s set of bone maladies is unique in several respects. The specimen bears, by a substantial margin, the greatest known number and variety of pathological features of the pectoral girdle and forelimb in a theropod dinosaur. It also is the only known dinosaur specimen with fractures in the pectoral girdle and the ipsilateral forelimb; with maladies in an ipsilateral radius and ulna; with maladies in both radii; with fibriscesses in both the forearm and the hand; and with evidence of possible osteodysplasia in a limb. The specimen is therefore, in several respects, a superlative example of dinosaur paleopathology.

It is not possible to determine with certainty the number of traumatic events that this plethora of pathological features represents. It is possible that the entire array of fractures and punctures is the result of a single, high-energy encounter; for example, the fractures may have been caused by collision with a tree or a rock wall during a fight in which a conspecific or a prey item caused the puncture wounds with hand and/or toe claws. It is at least certain that the animal survived the traumatic event(s), because the high degree of healing of all fractures and fibriscesses indicates that the event(s) took place long before the animal’s death. During the healing period the ailments in their early states must have severely compromised the use of the forelimbs in prey capture. The survival of the animal despite these ailments therefore suggests a prolonged period of fasting or subsisting on prey small enough to be dispatched with the mouth and/or feet alone or with the use of only one forelimb. It is also a testament to the hardiness of an animal that doubtlessly experienced an agonizingly long duration (or durations) of high degrees of pain in multiple locations.

Pathological features in dinosaur skeletons are underreported. Researchers describing new dinosaur species often make no mention of pathological features that are present in skeletons that they are describing. The holotype of *Dilophosaurus wetherilli* is an example of such omission. The specimen’s original and otherwise-thorough description [12] mentions only one of the pathological features reported here, the fibriscessed left thumb phalanx. At least in some cases, the motivation behind such omission seems to be the principle that a description of a new species should include only those traits characteristic of the species and not aberrant traits [5]. In other cases the pathological feature may simply be deemed unimportant or may not be recognized. Osteodysplasia may be particularly difficult to recognize because of the similarity between developmental deformity and taphonomic distortion.

Bone abnormalities in dinosaur skeletons may not contribute useful information for species diagnoses, but they do elucidate aspects of the lives of the specimens in question. This specimen, for example, underwent—and survived—severe trauma. Our study therefore underscores the need to reexamine described specimens in order to reconstruct details of their life histories that only an examination of pathological features can provide.
